# 外泌体在肺癌诊断及治疗中的研究进展

**DOI:** 10.3779/j.issn.1009-3419.2016.11.10

**Published:** 2016-11-20

**Authors:** 洪波 邹, 红 邬, 川 许

**Affiliations:** 1 646000 泸州，西南医科大学附属医院 The Affiliated Hospital of Southwest Medical University, Luzhou 646000, China; 2 610083 成都，成都军区总医院肿瘤诊治中心 Department of Oncology, Chengdu Military General Hospital, Chengdu 610083, China; 3 530021 南宁，广西医科大学 Guangxi Medical University, Nanning 530021, China

**Keywords:** 肺肿瘤, 外泌体, 肿瘤发生与演进, 诊断, 治疗, Lung neoplasms, Exosomes, Tumor initiation and progression, Diagnosis, Treatment

## Abstract

肺癌是全球范围内发病率及死亡率最高的恶性肿瘤，其严重威胁着人类健康。外泌体（exosomes）是起源于多泡体的纳米级脂质膜囊泡，其内含有蛋白质、脂质、核酸等多种活性生物分子。外泌体在肺癌的发生与演进中发挥重要作用，其可促进肺癌微环境形成，增强肿瘤侵袭与转移能力，参与肿瘤免疫抑制及肿瘤放化疗抵抗，且对肺癌的早期诊断和治疗具有应用价值。本文将对外泌体在肺癌发生、发展、诊断及治疗中的研究进展进行综述。

## Abstract (en)

As the leading cause of morbidity and cancer related-death worldwide, lung cancer has a serious threat to human health. Exosomes are nanoscale lipid membrane vesicles derived from multivesicles, which containing active biomolecules including proteins, lipids, nucleic acids and *etc*. Exosomes play important roles in lung cancer initiation and progression by promoting the formation of tumor microenvironment, enhancing tumor invasive and metastasis capability, leading to immunosuppression and resistance to chemoradiotherapy, and also have the application value in early diagnosis and treatment. This review summarizes the research progress of exosomes in tumor initiation and progression, and its roles in diagnosis and treatment of lung cancer.

1983年Johnstone等^[1]^在研究网织红细胞成熟的过程中发现了一种和转铁蛋白受体相关的膜型囊泡（vesicles）被分泌到细胞外，并在1987年将这种在超速离心下获得的囊泡命名为外泌体。此后，有研究^[2]^发现外泌体中含有蛋白质、脂质、核酸等多种成分，并在多个生理和病理过程中发挥重要作用。近年来，外泌体在肿瘤中的研究如火如荼。大量研究发现，外泌体可通过促进肺癌微环境形成，增加肿瘤细胞侵袭与转移能力，介导肿瘤免疫抑制，参与放化疗抵抗等促进肺癌的发生与发展。研究外泌体在肿瘤发生与发展中的作用机制可为肺癌早期有效的诊断和治疗提供新的思路。本文对外泌体在肺癌发生发展、诊断及治疗中的研究进展进行综述。

## 1 外泌体概论

### 1.1 外泌体的生物学特点和成分

外泌体是源于细胞“内吞-融合-外排”等调控过程而形成的胞外纳米级多泡体（multivesicular bodies, MVBs），在电子显微镜下外泌体呈现典型的50 nm-100 nm杯状或蝶形脂质双分子膜结构^[3]^，蔗糖梯度离心显示其密度约为1.13 g/mL-1.21 g/mL^[4]^。利用超速离心法、密度梯度离心法、色谱法、免疫磁珠法等方法可在正常细胞或肿瘤细胞培养基上清液、血液、唾液中检测并分离、纯化外泌体^[5, 6]^。由于外泌体起源细胞不同且成分复杂，目前尚无标准的分子标记物，但有学者^[7]^发现四次跨膜蛋白（CD9、CD63、CD81）、热休克蛋白70（heat shock protein 70, HSP70）、肿瘤易感基因101蛋白（tumor susceptibility gene 101, TSG101）、ALG-2相互作用蛋白X（ALG-2-interacting protein X, Alix）等可作为外泌体的标记物使用。目前已证实外泌体含有蛋白质、脂质、核酸等多种活性成分，其中蛋白质主要包含粘附蛋白[如：CD9、CD63、CD81、CD166、CD146、整合素）、信号转导蛋白（如：同线蛋白、网格蛋白）、分子伴侣（如：HSP70、HSP84）、抗原提成蛋白（如：主要组织相容性复合体I（major histocompatibility complex I, MHC I）和MHC II、CD86]等。脂质成分主要含有胆固醇、甘油二酯、鞘磷脂（包括神经鞘磷脂和神经酰胺）、甘油磷脂（包括卵磷脂、磷脂酰丝氨酸和磷脂酰乙醇胺）和饱和脂肪酸等。核酸中RNA除信使RNA（messenger RNA, mRNA）和微小RNA（microRNA, miRNA）外，还包括小干扰RNA（small interfering RNA）、穹窿体RNA（vault RNA）、转运RNA（transfer ribonucleic acid, tRNA）等非编码RNA（non-coding RNA, ncRNA）^[4, 8, 9]^；DNA除含有单链DNA（single-stranded DNA, ssDNA）和线粒体DNA（mitochondrial DNA）外，还包含双链DNA（double-stranded DNA, dsDNA）^[10, 11]^。

### 1.2 外泌体的生物学功能

研究发现，外泌体作为细胞外微环境的重要组成部分，可通过旁分泌等途径释放进入细胞外环境，并可通过以下途径在细胞间信号转导和物质传递中发挥重要作用：①外泌体能作为信号复合物通过受体介导直接刺激靶细胞；②外泌体可转运膜受体、细胞器、蛋白质、核酸等活性生物成分进入靶细胞内，通过影响靶细胞基因转录和翻译，调节细胞信号通路，从而改变受体细胞表型和影响细胞功能状态^[12, 13]^。研究^[14-16]^发现，外泌体不仅能抑制心肌缺血再灌注损伤，还能促进神经损伤后修复，并可通过促血管生成促进伤口愈合和组织修复。此外，外泌体还具有免疫调节等功能^[17]^。近年来研究^[18]^证据表明外泌体在肺癌的发生与发展中也扮演重要角色。

## 2 外泌体在肺癌发生与发展中的作用

肺癌是全球范围内发病率及死亡率最高的恶性肿瘤，其中非小细胞肺癌（non-small cell lung cancer, NSCLC）所占比例最高，约为85%^[18]^，其预后往往较差，5年生存率 < 15%^[19]^。外泌体作为肺癌细胞分泌的一种微型囊泡，在肺癌的发生、发展中发挥重要作用。其机制大致可概括为以下几点：①促进肺癌微环境形成以增加肿瘤细胞侵袭能力^[18]^。肿瘤细胞分泌的外泌体能通过降解细胞外基质（extracellular matrix, ECM）等形成肿瘤微环境，由于癌基因的不稳定性，使得低氧、酸中毒、饥饿在内的微环境诱导和炎症免疫反应等均能促进肿瘤细胞释放外泌体形成肿瘤微环境^[20]^。肿瘤微环境的形成有利于肿瘤细胞快速生长并增强其运动侵袭能力。Zhao等^[21]^发现肿瘤微环境中的肿瘤相关成纤维细胞能分泌含氨基酸、脂质和三羧酸循环中间产物等物质的外泌体，这些外泌体能在营养匮乏条件下为癌细胞生存提供必要营养成分。其次，促进肿瘤血管生成也可促进肺癌演进。Liu等^[22]^发现使用香烟提取物刺激支气管上皮细胞可激活STAT3信号通路，进而促使含miR-21的外泌体分泌增加，而含miR-21外泌体的增加将提高血管内皮生长因子（vascular endothelial growth factor, VEGF）水平从而促进肿瘤血管的生成并诱导支气管上皮细胞恶性转化。Cui等^[23]^还发现肺腺癌细胞能分泌含miR-210的外泌体调节基质细胞中酪氨酸受体激酶A3（ephrin A3）水平，进而促进肿瘤血管生成以维持肿瘤细胞的生长。此外，含Rab3D蛋白和转化生长因子-β（transforming growth factor beta, TGF-β）的外泌体能促进上皮细胞间叶化（epithelial-mesenchymal transition, EMT），增强肺癌细胞致瘤性和侵袭性^[24, 25]^。Wang等^[19]^在体外低氧条件下证实了肺癌细胞能增加含TGF-β和白细胞介素-10（interleukin-10, IL-10）的外泌体释放进入肿瘤微环境提高肺癌细胞运动迁移能力以促进其侵袭转移。②介导肺癌免疫抑制。研究发现大多数癌症患者存在免疫缺陷或免疫抑制，而肿瘤细胞能通过外泌体调节肿瘤免疫。其主要是通过携带免疫抑制因子并抑制免疫细胞（如树突状细胞、NK细胞、CD4^+^和CD8^+^ T淋巴细胞）发挥的抗肿瘤免疫效应，诱导免疫抑制和调节细胞群，例如髓源抑制性细胞（myeloid-derived suppressor cells, MDSCs）、调节性T细胞（Tregs）、调节性B细胞（Bregs）等^[26, 27]^，而MDSCs具有抑制T细胞免疫应答能力^[17]^。Chalmin等^[28]^发现肺腺癌细胞分泌的含HSP72外泌体能激活STAT3信号通路从而介导MDSCs的免疫抑制作用。此外，外泌体也能通过转导特定蛋白和RNA进入受体细胞发挥免疫逃逸作用^[29]^。③参与肺癌放化疗抵抗。肺癌化疗耐药和放疗抵抗是肺癌治疗失败的主要原因，研究发现外泌体能作为肿瘤细胞排出化疗药物（尤以铂类和阿霉素类为主）的主要形式而产生化疗耐药，从而大大降低化疗疗效^[30]^。Xiao等^[31]^发现接触顺铂的肺癌细胞能分泌大量的外泌体沟通其他肺癌细胞并增加其对顺铂的耐药性，而Li等^[32]^则发现使用吉非替尼处理肺癌细胞后产生的外泌体能拮抗顺铂的化疗疗效。Choi^[33]^及Jung等^[34]^也通过蛋白质组学分析发现肺癌细胞分泌的外泌体中多种蛋白质成分和磷脂成分参与吉非替尼的耐药性。此外，外泌体也介导放疗抵抗。Tang等^[35]^发现采用X射线进行肺癌放疗将诱使含miR-208a外泌体增加，而miR-208a的增加可通过锚定*p21*基因激活AKT/mTOR信号通路，进而导致肺癌放疗抵抗并促进肺癌细胞增殖。此外，Yuan等^[36]^发现miR-1246能通过锚定死亡受体5（death receptor 5, DR5）增强肺癌细胞放疗抵抗。因此，外泌体可通过多途径参与肺癌的发生演进。

## 3 外泌体在肺癌诊断中的应用

尽管目前低剂量CT（low-dose computed tomography, LDCT）在肺癌高危人群筛查上取得了一定的成就^[37]^，但仍缺乏精确有效的早期筛查方法，多数患者确诊时已是癌症晚期，其5年生存率极低。研究发现，外泌体不仅与肺癌的发生演进密切相关，而且在诊断中也发挥着重要作用：①外泌体RNA在肺癌诊断中的作用。肺癌外泌体RNA水平明显高于健康者，且RNA水平与肺癌的进展程度、治疗反应、生存率都密切相关^[38]^。通过基因芯片、高通量测序法和定量即时聚合酶链锁反应（quantitative real time polymerase chain reaction, qRT-PCR）等方法发现血液循环中的miRNA等对NSCLC的诊断有较高价值^[39]^。其中Roth等^[40]^发现血液中的miR-361-3p和miR-625^*^有助于从良性肺部病变中识别恶性肺癌。而血液中hsa-miR-21的上调是早期诊断鳞状细胞肺癌的可靠生物学标志^[41]^，肺腺癌患者血液中miR-200b-5p、miR-378、miR-502-5p、miR-629、miR-17和miR-100较肺部肉芽肿患者和健康吸烟者明显增高^[42]^。此外，Rodriguez等^[43]^发现肺部肿瘤患者血液中外泌体含有的miR-122-5p等miRNA含量明显高于支气管肺泡灌洗液。Munagala等^[44]^发现肺癌外泌体中miR-21和miR-155升高对于肺癌复发诊断是潜在的生物学标志。②外泌体蛋白质在肺癌诊断中的作用。肺癌细胞分泌的外泌体中富集多种蛋白质成分并促进肺癌发生演进，是早期诊断肺癌的有效途径^[45]^。通过肺癌细胞分泌的细胞外囊泡分析发现，外泌体表面高表达CD317、表皮生长因子受体（epidermal growth factor receptor, EGFR）等，这些分子均为诊断NSCLC的可靠生物学标志^[46, 47]^。此外，Li等^[48]^发现检测尿液中外泌体的人富亮氨酸α2糖蛋白1（human leucine-rich α2-glycoprotein 1, LRG1）是诊断NSCLC患者的潜在标记物。综上所述，关注外泌体及其相关成分可为探索肺癌早期诊断的分子标志物提供理论依据。

## 4 外泌体在肺癌治疗中的作用

目前，肺癌的治疗以手术、化学治疗、放射治疗、分子靶向治疗的综合治疗为主，但由于各种治疗方式均存在局限性及肺癌患者易发生早期转移及复发的特点，导致患者预后往往较差。研究发现，基于外泌体及其相关成分的研究可为肺癌患者的治疗带来新希望。其依据大致可概括为以下几点：①外泌体可增强化疗药物敏感性。Li等^[49]^发现肺腺癌中miR-181a能通过锚定人第10号染色体缺失的磷酸酶及张力蛋白同源基因（phosphatase and tensin homolog deleted on chromosome ten, PTEN）抑制EMT，并能增加肺腺癌细胞对紫杉醇和铂类化疗敏感性。通过使用去乙酰化酶抑制剂（如Trichostatin A）和去甲基化药物（如5´aza-deoxycytidine plus）可使表观沉默的miR-512和miR-373活化，继而增加肺癌细胞对铂类的化疗敏感性^[50]^。Aqil等^[51]^发现将从植物中萃取的三萜烯化合物雷公藤红素装载于外泌体中能抑制肺癌细胞增殖，并增加化疗敏感性和减少毒副作用。②开发肿瘤疫苗。肿瘤疫苗是一种活性生物前体，而外泌体目前已成为肿瘤疫苗研究的热点。肺癌细胞相关抗原刺激树突状细胞等抗原提成细胞产生携带有特异癌抗原的外泌体^[52]^，当外泌体迁移到区域淋巴结，可激活CD4^+^和CD8^+^ T淋巴细胞产生强大的抗肿瘤免疫反应。因此，以外泌体为基础的肿瘤疫苗可为肺癌治疗提供新的思路^[53]^。例如：Ⅲ期临床试验证实治疗性疫苗（belagenpumatucel-L）^[54]^和TG4010^[55]^治疗可延长NSCLC晚期患者的生存期。此外，脂质体疫苗BLP-25^[56]^和表皮生长因子疫苗（EGF vaccination）^[57]^和人类黑色素瘤抗原-A3疫苗（MAGE-A3 vaccine）^[58]^对于NSCLC治疗也具有一定疗效。③降低外泌体含量。由于肺癌细胞相关外泌体介导肺癌发生与演进，采用血液过滤系统（如Aethlon ADAPT^TM^ system）去除或减少外周循坏中外泌体含量可为肺癌治疗提供新方法^[59]^。Fabbri等^[60]^发现使用中性鞘磷脂酶抑制剂GW4869能抑制小鼠体内外泌体的产生，并减少肺癌的转移。④外泌体可抑制原癌基因表达。研究发现通过外泌体抑制肺癌相关原癌基因表达将有效抑制肺癌的发生进展。有研究^[61, 62]^发现使用RNA干扰技术（RNA interference, RNAi）能使肺癌相关原癌基因（如：*AKT1*、*WT1*、*IGF-1R*、*NUPR1*、*LMO3*）沉默，并促使肺癌相关mRNA降解。因此，通过外泌体抑制肺癌相关致癌基因表达将有效抑制肺癌的发生。⑤外泌体可促进肺癌细胞凋亡。由于肺癌细胞能通过外泌体分泌生存素抑制肺癌细胞凋亡和促进肺癌细胞生长，使用生存素基因负性突变体（Survivin-D53A）可促进肺腺癌细胞凋亡，而成为潜在的基因治疗药物^[63]^。此外，有学者发现从姜科植物中提取的β-榄香烯（β-Elemene）能促进抑癌基因*p53*活化并促进肺癌细胞凋亡^[64]^。Ma等^[65]^发现miR-34a能通过锚定转化生长因子β受体2（transforming growth factor beta receptor II, TGFβR2）抑制肺癌细胞增殖并促进其凋亡。⑥外泌体可作为肺癌潜在的治疗靶点。肺癌相关外泌体成分和功能的多样性为肺癌的治疗提供多个潜在治疗靶点。如：黑色素瘤相关基因（mda-9/syntenin）过表达将促进小细胞肺癌释放外泌体，通过以黑色素瘤相关蛋白为靶点设计新药将有可能成为肺癌治疗新方法^[66]^。针对Ⅱ型跨膜丝氨酸蛋白4（transmembrane protease, serine 4, TMPRSS4）的封闭肽或抗体能促使miR-205高表达和抑制整合素α5水平从而有效降低肺癌的侵袭转移^[67]^。此外，有学者^[24]^发现从蔷薇科植物中提取的地榆素H6能通过抑制TGF-β的活性而抑制肺腺癌的进展。Yang等^[68]^也发现促进外泌体中let-7的表达是肺癌治疗的潜在靶点。因此，围绕肺癌相关外泌体的治疗研究将为探索肺癌个体化、精准化治疗策略提供新思路。

## 5 展望

肺癌是威胁人类健康的重大恶性疾病，尽管人们已认识到外泌体在肺癌的发生、发展中发挥重要作用，也围绕外泌体在肺癌的早期诊断和治疗领域中进行了大量探索性工作，但外泌体作为肿瘤微环境的重要组成部分在肿瘤演进中的具体作用机制尚不清楚，外泌体应用于肺癌诊断和治疗的敏感性和特异性仍有待提高。因此，关注外泌体在肿瘤演进中的作用机制及转化医学研究将为肺癌早期诊断和治疗提供更丰富的理论依据和参考价值。
